# Chronic postoperative pain after total knee arthroplasty: The potential contributions of synovitis, pain sensitization and pain catastrophizing—An explorative study

**DOI:** 10.1002/ejp.2018

**Published:** 2022-08-19

**Authors:** Thomas Kurien, Robert W. Kerslake, Thomas Graven‐Nielsen, Lars Arendt‐Nielsen, Dorothee P. Auer, Kimberley Edwards, Brigitte E. Scammell, Kristian Kjær‐Staal Petersen

**Affiliations:** ^1^ University of Nottingham Academic Orthopaedics, Trauma and Sports Medicine Nottingham UK; ^2^ Nottingham Elective Orthopaedic Service (NEOS) Nottingham University Hospitals NHS Trust Nottingham UK; ^3^ Pain Centre Versus Arthritis University of Nottingham Nottingham UK; ^4^ NIHR Nottingham Biomedical Research Centre University of Nottingham Nottingham UK; ^5^ Sir Peter Mansfield Imaging Centre University of Nottingham Nottingham UK; ^6^ Center for Neuroplasticity and Pain (CNAP), Faculty of Medicine Aalborg University Aalborg Denmark; ^7^ Mech‐Sense, Department of Gastroenterology and Hepatology Aalborg University Hospital Aalborg Denmark; ^8^ Center for Mathematical Modelling of Knee Osteoarthritis, Department of Materials and Production Aalborg University Aalborg Denmark

## Abstract

**Background:**

A subset of osteoarthritis patients will experience chronic postoperative pain after total knee arthroplasty (TKA), but the source of pain is unclear. The aim of this exploratory study was to assess patients with and without postoperative pain after TKA using magnetic resonance imaging (MRI), quantitative sensory testing (QST), clinical assessment of pain and assessments of catastrophizing thoughts.

**Methods:**

Forty‐six patients completed the 6‐month postoperative assessment. MRI findings were scored according to the MRI Osteoarthritis Knee Score recommendation for Hoffa synovitis, effusion size and bone marrow lesions. QST included assessment of pressure pain thresholds (PPTs), temporal summation of pain (TSP) and conditioned pain modulation (CPM). Pain catastrophizing was assessed using the Pain Catastrophizing Scale (PCS). Clinical pain assessment was conducted using a visual analogue scale (VAS, 0–10 cm), and groups of moderate‐to‐severe (VAS > 3) and none‐to‐mild postoperative pain (VAS ≤ 3) were identified.

**Results:**

Patients with moderate‐to‐severe postoperative pain (*N* = 15) demonstrated higher grades of Hoffa synovitis (*p* < 0.001) and effusion size (*p* < 0.001), lower PPTs (*p* = 0.039), higher TSP (*p* = 0.001) and lower CPM (*p* = 0.014) when compared with patients with none‐to‐mild postoperative pain (*N* = 31). No significant difference was found in PCS scores between the two groups. Multiple linear regression models found synovitis (*p* = 0.036), effusion size (*p* = 0.003), TSP (*p* = 0.013) and PCS (*p* < 0.001) as independent parameters contributing to the postoperative pain intensity.

**Conclusion:**

These exploratory findings could indicate that chronic postoperative pain after TKA is a combination of joint‐related synovitis and effusion, sensitization of central pain mechanisms and potentially pain catastrophizing thoughts, but larger studies are needed to confirm this.

**Significance:**

The end‐stage treatment of knee osteoarthritis is total knee arthroplasty. Some patients experience chronic postoperative pain after total knee arthroplasty, but the mechanism for chronic postoperative pain is widely unknown. The current study indicates that higher levels postoperative of synovitis and effusion, higher temporal summation of pain and higher pain catastrophizing scores could be associated with higher chronic postoperative pain.

## 1 INTRODUCTION

Total knee arthroplasty (TKA) is the end‐stage treatment for osteoarthritis (OA), and a subset of patients experience chronic postoperative pain after TKA (Beswick et al., 2012). Revision TKA surgery is associated with a high risk of worsening the postoperative pain (Petersen, Simonsen, et al., 2015) and the treatment options for chronic postoperative pain are limited. Although pain in OA is well studied, postoperative factors contributing to chronic postoperative pain after TKA is less studied, and therefore, the treatment of postoperative pain after TKA is difficult. It seems evident that revision TKA should not be performed on the indication of pain, but identifying postoperative factors that promote chronic postoperative pain might provide treatment targets in the future.

OA pain is a combination of joint‐specific factors and non‐joint‐specific factors with bone marrow lesions (BMLs), knee effusion and synovitis examples of joint‐specific factors, which amplify OA pain (Felson, 2005; Guermazi et al., 2012, 2014; Hill et al., 2001). Non‐joint‐specific factors can include cognitive factors, such as pain catastrophizing, and sensitization of the nervous system. Pain catastrophizing thoughts are present in a subset of patients with OA (Edwards et al., 2011) and are associated with increased clinical pain intensity (Quartana et al., 2009). Higher levels of preoperative pain catastrophizing scores are a known predictor of chronic postoperative pain after TKA (Larsen, Laursen, Edwards, et al., 2021; Theunissen et al., 2012), but the association between postoperative pain catastrophizing and chronic postoperative pain after TKA has not been studied. Sensitization of the central nervous system can be manifested as widespread pressure hyperalgesia, which can be assessed using pressure pain thresholds (PPTs) (Arendt‐Nielsen & Graven‐Nielsen, 2011). A prolonged peripheral pain input can lead to increased sensitivity of dorsal horn neurons (Schaible, 2004), which in humans can be assessed by as temporal summation of pain (TSP) (Arendt‐Nielsen & Graven‐Nielsen, 2011). Descending pain inhibitory control systems is another important modulator of pain, and conditioned pain modulation (CPM) is believed to assess one of these pathways in humans (Yarnitsky, 2010). Lower PPTs, facilitated TSP and impaired CPM is often found in patients with severe OA when compared to healthy pain‐free individuals (Arendt‐Nielsen, Skou, et al., 2015) and this could indicate that these patients are pain sensitive. These measurements have been associated with poor outcomes after standard OA pain therapies such as exercise‐based therapy (Hansen et al., 2020), weeks of non‐steroidal anti‐inflammatory drugs (Arendt‐Nielsen et al., 2016; Edwards et al., 2016; Petersen et al., 2021; Petersen, Olesen, et al., 2019; Petersen, Simonsen, et al., 2019) and TKA (Kurien et al., 2018; Petersen et al., 2016; Petersen, Arendt‐Nielsen, et al., 2015). Pain‐alleviating TKA surgery seems to normalize PPTs, TSP and CPM (Graven‐Nielsen et al., 2012; Kosek & Ordeberg, 2000), but conflicting studies exists (Petersen, Arendt‐Nielsen, et al., 2015).

Since chronic postoperative pain after TKA is a major clinical issue, this exploratory study aimed to assess patients with and without pain 6 months after TKA using MRI of the knee, experimental pain profiling (PPT, TSP, CPM), clinical assessment of pain and assessment of catastrophizing thoughts to explore potential factors associated with chronic postoperative pain.

## 2 METHODS

### 2.1 Participants

A consecutive series of patients scheduled for primary TKA at orthopaedic clinics in Nottingham, United Kingdom, were recruited, and the results from the preoperative associations to chronic postoperative pain have already been published (Kurien et al., 2018). All patients underwent unilateral TKA due to knee OA as assessed by the American College of Rheumatology Criteria (Altman et al., 1990). Knee radiographs were obtained from the patients with OA (anterior–posterior, lateral and skyline views) as part of the routine preoperative care and were graded using the Kellgren–Lawrence system for OA (Kellgren & Lawrence, 1957). The patients were recruited 6 months postoperatively if they had not undergone any previous surgery to the knee (other than the TKA studied here), were over 40 years of age and were able to give informed consent. Exclusion criteria included symptomatic hip OA, major psychiatric or neurological illness, active cancer, sensory dysfunction, contraindication to MRI, other chronic pain condition, for example, fibromyalgia, rheumatoid arthritis or requiring opioid or neuropathic analgesia (e.g., pregabalin, gabapentin). The study was approved by East Midlands Nottingham ethics committee (REC reference: 10/H0408/115) and the University of Nottingham ethics committee (Ethics Ref: H11122014) and was carried out under the principles of the Declaration of Helsinki. The study was registered on clinicaltrials.gov (NCT03126279). All participants gave informed consent.

### 2.2 Protocol

All patients were invited for a 6‐month postoperative follow‐up visit and all patients underwent MRI of the knee with the implanted prosthesis and assessments of PPTs, TSP and CPM. Furthermore, the patients completed the Oxford Knee Score and the Pain Catastrophizing Scale (PCS). Postoperative pain was assessed as the worst pain within the last 24 h using a visual analogue scale (VAS, 0–10 cm). Based on the pain assessment, the patients were divided into patients with moderate‐to‐severe (VAS > 3 cm) or none‐to‐mild postoperative pain (VAS ≤ 3 cm).

PCS consists of 13 items focusing on thoughts and feelings in connection with pain (Sullivan et al., 1995). The questions are rated on a 4‐point scale ranging from 0 (not at all) to 3 (very much). The overall score is the sum of all items and can range from 0 to 52, with higher scores corresponding to more pain catastrophizing thoughts.

The Oxford Knee Score (OKS) consists of 12 questions that cover function and pain of the knee. Each question is scored from 0 to 4 (0 being the worst outcome and 4 being the best). The overall score is the sum of all items and can range from 0 to 48, with higher scores corresponding to better outcomes.

#### 2.2.1 Magnetic resonance imaging of the prosthesis‐implanted knee

The patients underwent a dedicated knee MRI at 3.0 Tesla (GE MR750, GE Healthcare, Waukesha, WI) using a 3D multi‐acquisition variable resonance image combination selective (MAVRIC SL®) sequence to reduce metal artefacts and an 8‐channel phased array transmit–receive knee coil to assess BMLs, synovitis or effusions around the prosthesis. Images were acquired in coronal, sagittal and axial planes using intermediate‐weighted (MAVRIC PD) and inversion recovery fat‐suppression (MAVRIC STIR) sequences. Image parameters for MAVRIC PD were slice thickness 4 mm (no gap), repetition time/echo time 3300/7.4 ms, echo train length (ETL) 20, pixel bandwidth 488 Hz/pixel, reconstruction diameter 180 mm, acquisition matrix 320 × 256, flip angle 85 degrees and 0.5 excitations. For MAVRIC STIR, parameters were slice thickness 4 mm (no gap), repetition time/echo time 4300–5000/7.0 ms, ETL 20, pixel bandwidth 976 Hz/pixel, reconstruction diameter 180 mm, acquisition matrix 256 × 192, flip angle 75 degrees and 0.5 excitations. The images were scored independently by a senior musculoskeletal radiologist and an orthopaedic surgeon for postoperative BMLs (0–18), effusion synovitis (0–3) and Hoffa synovitis (0–3) using the standard MRI Osteoarthritis Knee Score (MOAKS) criteria (Hunter et al., 2011). Along with a large femoral drill tract present in all cases due to intraoperative intramedullary referencing for femoral sizing and with a significant proportion of the femoral surface being replaced by the implant, the tibial region alone was scored for post‐TKA BMLs. Six out of the seven MOAKS scores of the tibial sub‐regions were used to assess BMLs. The subspinous sub‐region was excluded as the majority of this region was occupied by the tibial stem of the prosthesis in all cases (Hunter et al., 2011). An overall total BML score was calculated (0–18) for the tibia by summing the BML scores in the individual six sub‐regions, with higher scores being indicative of increasing BMLs.

#### 2.2.2 Pain sensory profile

Pain sensory profiles were evaluated by cuff pressure stimuli using a computer‐controlled cuff algometer (Cortex Technology and Nocitech, Aalborg University) including a 13‐cm wide tourniquet cuff (VBM) and an electronic VAS (Aalborg University) for recording of the pain intensity. The sampling rate of the pressure and VAS data was 25 Hz. The cuff was placed at the level of the head of the gastrocnemius muscle of the leg, which previous received the TKA. The electronic continuous VAS was 10‐cm long and sampled at 10 Hz; 0 cm indicated ‘no pain’ and 10 cm indicated ‘maximum pain’.

Cuff PPTs were assessed bilaterally by means of 1 kPa/s pressure increases. The patients were instructed to rate the pain intensity continuously on the electronic VAS as the pressure increased. The pain tolerance threshold was defined as the time point when the pressure pain became intolerable, which the patients were to indicate by pressing the ‘stop button’. The PPT was defined as the pressure at which the VAS score exceeded 1 cm as previously used (Graven‐Nielsen et al., 2015; Imai et al., 2016; Kurien et al., 2018; Rathleff et al., 2015; Vaegter & Graven‐Nielsen, 2016).

TSP was assessed by providing 10 short‐lasting stimuli (1 s each) at the level of the pressure pain tolerance threshold with a 1‐s break between stimuli. The participants were instructed to continuously rate the pain intensity of the sequential stimuli using the electronic VAS and not to return to zero during the breaks. For each cuff stimulus, a VAS score was extracted and TSP was defined as the difference between the tenth and the first VAS score (Petersen, Arendt‐Nielsen, et al., 2015).

CPM was assessed as the changes in cuff PPT with and without a conditioning stimulus (cuff pressure stimulation) on the contralateral leg. This combination has proven reliable (Graven‐Nielsen et al., 2017; Imai et al., 2016). The conditioning stimulus was applied in parallel to the test stimulus as a constant stimulus with an intensity of 70% of the pain tolerance level (Graven‐Nielsen et al., 2017). This CPM protocol has previously been used when studying patients with chronic pain (Heredia‐Rizo et al., 2019; Holden et al., 2018; Izumi et al., 2017; Kurien et al., 2018; Petersen et al., 2020; Rathleff et al., 2015). The CPM effect was calculated as the absolute difference in PPT with and without a conditioned stimulus.

The cuff assessments were completed once, since previous reliability studies demonstrate good‐to‐excellent reliability of these assessments (Graven‐Nielsen et al., 2015; Imai et al., 2016).

### 2.3 Statistical analysis

The data are presented as means and standard deviation (SD), if not otherwise stated. Independent *t* tests were used to compare demographic data, postoperative MRI findings, PCS and the pain sensory profiling findings comparing patients with moderate‐to‐severe and none‐to‐mild chronic postoperative pain and Cohen's *d* was reported as a measure of effect size. Chi‐square tests were used to assess gender differences and differences in KL scores when comparing the two groups. Pearson's correlations (one‐tailed) were applied to investigate associations between pain sensory profiles, pain catastrophizing and MRI findings and these correlations were not adjusted for multiple comparison. Linear regression analyses, with chronic postoperative pain VAS scores as the dependent variable and postoperative MRI findings, cognitive factors and pain sensory profiling findings as independent variables, were used to explain factors associated with chronic postoperative pain. The backwards elimination model was applied to the linear regression models to find independent factors for chronic postoperative pain. The adjusted *R* ^2^ are reported for all linear regression models as a measure of the models' ability to explain the postoperative pain intensity. The statistical analyses were conducted using SPSS (version 27, IBM Corporation, New York, USA). *p* values <0.05 were considered significant. The current work is a secondary analysis from Kurien et al. (2018), and therefore, the results should be considered exploratory.

## 3 RESULTS

For this exploratory analysis, 46 patients had complete postoperative data with 15 patients being categorized as ‘moderate‐to‐severe postoperative pain’ and 31 patients being categorized as ‘none‐to‐mild postoperative pain’ (see Table 1 for demographics). The groups were not significantly different when comparing preoperative age, body mass index (BMI), gender distribution, Kellgren–Lawrence score, pain duration or pain catastrophizing thoughts. The moderate‐to‐severe postoperative pain group demonstrated significantly higher preoperative pain scores (*t* test: *p* = 0.008) and a trend towards lower preoperative OKS scores (*t* test: *p* = 0.085).

**TABLE 1:** Demographics (mean and SD) of patients with moderate‐to‐severe and none‐to‐mild postoperative pain assessed 6 months after primary total knee arthroplasty.

	Moderate‐to‐severe pain	None‐to‐mild pain	*p*
	(*N* = 15)	(*N* = 31)	
Age (years)	68.0 (SD: 7.2)	66.9 (SD: 8.4)	0.667
BMI (kg/m^2^)	29.9 (SD: 3.4)	29.7 (SD: 4.9)	0.911
Gender (% females)	60%	65%	0.766
Preoperative Kellgren–Lawrence score (0–4)	3 (73%) 4 (27%)	3 (45%) 4 (55%)	0.138
Preoperative VAS (0–10)	6.6 (SD: 2.1)	4.6 (SD: 2.4)	**0.008**
Preoperative pain duration (months)	43.8 (SD: 34.4)	46.3 (SD: 26.7)	0.787
Preoperative Oxford Knee Score (0–48)	19.4 (SD: 7.0)	23.5 (SD: 7.8)	0.085
Postoperative Pain Catastrophizing Scale (0–52)	10.9 (SD: 11.8)	6.1 (SD: 8.7)	0.128

Bold indicate significant findings.

Abbreviations: BMI, body mass index; SD, standard deviation; VAS, visual analogue scale.

### 3.1 Postoperative MRI findings

**FIGURE 1:**
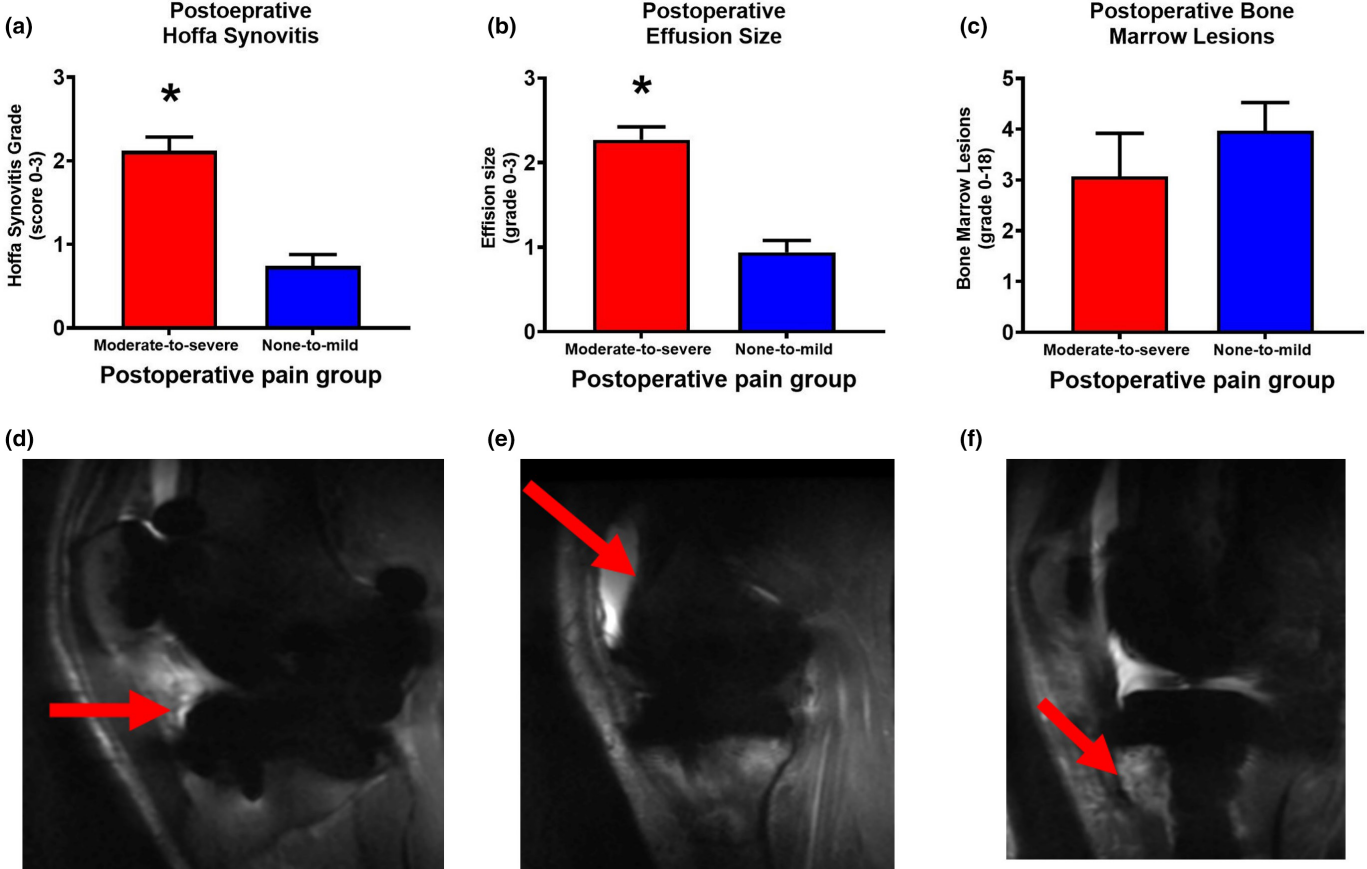
Joint magnetic resonance imaging (MRI) findings from patients with moderate‐to‐severe (red boxes) and none‐to‐mild (blue boxes) postoperative pain 6 months after total knee arthroplasty. The MRI Osteoarthritis Knee Score (MOAKS) was used to score (a) Hoffa synovitis findings, (b) effusion size and (c) bone marrow lesions [postoperative bone marrow lesions are graded from 0 to 18 (and not from 0 to 45 as per recommendations; Hunter et al., 2011) with only the tibia being included, as the entire femur was replaced by the femoral implant] (mean and SD). Examples of postoperative (d) Hoffa synovitis, (e) effusion and (f) bone marrow lesions. *p < 0.05.

Patients with moderate‐to‐severe postoperative pain demonstrated a higher grade of postoperative Hoffa synovitis (*t* test: *p* < 0.001, Cohen's *d*: 1.897) and a higher degree of postoperative effusion size (*t* test: *p* < 0.001, Cohen's *d*: 1.849) as compared with patients with none‐to‐mild postoperative pain when assessed 6 months postoperatively (Figure 1). The BMLs were not significantly different when comparing the two groups (*p* = 0.369).

### 3.2 Postoperative pain mechanistic pain profiles

**FIGURE 2:**
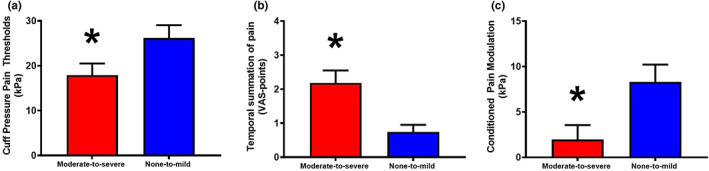
Pain sensory profiles from patients with moderate‐to‐severe (red) and none‐to‐mild (blue) chronic postoperative pain 6 months after total knee arthroplasty (mean and SD). The cuff algometer was used to assess cuff (a) pressure pain thresholds, (b) temporal summation of pain and (c) conditioned pain modulation. *p < 0.05. VAS, visual analogue scale.

Patients with moderate‐to‐severe postoperative pain reported lower postoperative PPT (*t* test: *p* = 0.039, Cohen's *d*: 0.574), facilitated postoperative TSP (*t* test: *p* = 0.001, Cohen's *d*: 1.159) and impaired postoperative CPM (*t* test: *p* = 0.014, Cohen's *d*: 0.676) when compared with patients with none‐to‐mild postoperative pain (Figure 2).

### 3.3 Postoperative pain catastrophizing

No statistical differences were found in PCS scores when comparing patients with moderate‐to‐severe postoperative pain (mean: 10.9 [SD: 11.8]) and patients with none‐to‐mild postoperative pain (mean: 6.1 [SD: 8.7], *p* = 0.128, Cohen's *d*: 0.489).

### 3.4 Correlations between postoperative pain, postoperative MRI, pain catastrophizing and pain sensory profiles

Significant Pearson's correlations (one‐tailed) were found between postoperative pain and postoperative PPT (*r* = −0.308, *R* ^2^ = 0.09, *p* = 0.019), TSP (*r* = 0.534, *R* ^2^ = 0.285, *p* < 0.001), CPM (*r* = −0.314, *R* ^2^ = 0.010, *p* = 0.017), postoperative Hoffa synovitis grade (*r* = 0.603, *R* ^2^ = 0.364, *p* < 0.001), postoperative effusion size grade (*r* = 0.594, *R* ^2^ = 0.353, *p* < 0.001) and postoperative PCS (*r* = 0.398, *R* ^2^ = 0.158, *p* = 0.003), but not postoperative BML (*r* = −0.181, *R* ^2^ = 0.033, *p* = 0.115).

Significant Pearson's correlations (one‐tailed) were found between postoperative TSP and postoperative PPT (*r* = −0.306, *R* ^2^ = 0.094, *p* = 0.019), postoperative CPM (*r* = −0.351, *R* ^2^ = 0.123, *p* = 0.008), postoperative Hoffa synovitis grade (*r* = 0.560, *R* ^2^ = 0.314, *p* = 0.028), postoperative effusion size grade (*r* = 0.561, *R* ^2^ = 0.315, *p* = 0.027) and postoperative PCS (*r* = 0.247, *R* ^2^ = 0.061, *p* = 0.049). No correlations were observed between the remaining combinations of parameters.

### 3.5 Factors associated with postoperative pain

Linear regression models using postoperative pain parameters were established to investigate factors associated with postoperative pain. Model 1 included all postoperative pain parameters and explained 64.5% of the variance (adjusted *R* ^2^ = 0.645). Following four iterations of backwards eliminations of model 1, the final model (model 2) explained 65.3% of the variance (adjusted *R* ^2^ = 0.653) with postoperative PCS, TSP, Hoffa synovitis grade and effusion grade as postoperative independent parameters (Table 2).

**TABLE 2:** Linear regression models aiming to explain factors associated with chronic postoperative pain 6 months after total knee arthroplasty.

Model	Postoperative parameters	Standardized coefficient	*p*	Adjusted *R* ^2^
1				**0.645**
	Cuff pressure pain threshold	0.105	0.474	
	Temporal summation of pain	0.245	**0.025**	
	Conditioned pain modulation	0.030	0.795	
	PCS scores	0.380	**<0.001**	
	Hoffa synovitis grade	0.276	**0.032**	
	Effusion grade	0.365	**0.008**	
	Bone marrow lesion^a^	−0.101	0.285	
2				**0.653**
	Temporal summation of pain	0.251	**0.013**	
	PCS scores	0.392	**<0.001**	
	Hoffa synovitis grade	0.263	**0.036**	
	Effusion grade	0.397	**0.003**	

*Note*: Clinical pain intensity was the dependent variable for all models and postoperative magnetic resonance imaging findings (synovitis, effusion and bone marrow lesions), pain sensory profiles (cuff pressure pain thresholds, temporal summation of pain and conditioned pain modulation) and pain catastrophizing were the independent parameters. Model 1 explained 64.5% of the postoperative pain intensity and model 2 explained 65.3% of the postoperative pain intensity following four iterations of backwards elimination. Bold indicate significant findings. Abbreviation: PCS, Pain Catastrophizing Scale.

^a^ Postoperative bone marrow lesions are graded from 0 to 18 (and not from 0 to 45 as per recommendations; Hunter et al., 2011) as only the tibia and patella were included as the entire femur was replaced by the femoral implant.

## 4 DISCUSSION

This exploratory study found that patients with moderate‐to‐severe chronic postoperative pain 6 months after TKA showed signs of synovitis and effusion around the prosthetic joint replacement together with decreased pressure pain thresholds in the lower leg, facilitated temporal summation of pain and impaired conditioned pain modulation compared with patients with none‐to‐mild chronic postoperative pain. Additionally, higher levels of postoperative temporal summation of pain, pain catastrophizing, synovitis scores and effusion scores were independently associated with higher levels of chronic postoperative pain. These data suggest that chronic postoperative pain after TKA is intensified by a combination of joint‐related factors, central pain sensitivity and pain catastrophizing, but larger studies are needed to confirm this.

### 4.1 Synovitis as a generator of pain

A recent systematic review and meta‐analysis found moderate evidence supporting the positive association between MRI‐detected synovitis and pain and conflicting evidence for a positive association between MRI‐detected effusion and pain in knee OA (Dainese et al., 2022). A subset of patients with knee OA demonstrate low‐grade systemic chronic inflammation (Siebuhr et al., 2014), and it is likely that synovitis can be a generator for such inflammation. Pro‐inflammatory cytokines, such as interleukin 6 (IL‐6), are known to sensitize peripheral nerve endings (Schaible, 2014), and one study has suggested that IL‐6 level is increased by synovitis and the presence of type 2 diabetes in patients with knee OA (Eitner et al., 2017).

A recent study has demonstrated that patients with moderate‐to‐severe chronic postoperative pain 5 years after TKA demonstrate elevated high‐sensitive C‐reactive protein (hsCRP) compared with patients with none‐to‐mild postoperative pain (Skrejborg et al., 2021). Studies indicate that synovitis is associated with elevated levels of hsCRP (Deng et al., 2022; Lin et al., 2022) and therefore the current study could suggest that the elevated hsCRP reported by Skrejborg et al. (2021) could, in part, arise from postoperative synovitis, but future studies should confirm this hypothesis. Furthermore, these results could indicate that increased levels of synovitis and effusion may be independently associated with increased chronic postoperative pain after TKA.

### 4.2 The contribution from cognitive factors on pain in osteoarthritis

It seems evident that psychological factors, such as pain catastrophizing, anxiety and depression, can facilitate clinical pain in osteoarthritis (Edwards et al., 2009). The serotonergic and noradrenergic systems play a critical role in the pathophysiology of anxiety (Dell'Osso et al., 2010; Gosmann et al., 2021) and depression (Furukawa et al., 2019). The Osteoarthritis Research Society International (OARSI) recommends duloxetine (a strong serotonin and noradrenaline re‐uptake inhibitor anti‐depressant) as a treatment option for patients with OA and/or widespread pain (Bannuru et al., 2019). Pain catastrophizing has often been found as a predictor of future pain (Kristensen et al., 2021; Larsen, Laursen, Edwards, et al., 2021; Petersen et al., 2020) and higher levels of pain catastrophizing, anxiety and depression are found in OA patients with poor quality of sleep when compared with patients with good quality of sleep (Larsen, Laursen, Simonsen, et al., 2021). A recent study on OA demonstrated that the combination of pre‐treatment QST, psychological factors and clinical pain could predict the analgesic effect of 14 weeks of duloxetine (Petersen et al., 2022), which underlines the importance of incorporating psychological factors in pain profiling of patients with OA. The current study indicates that postoperative pain catastrophizing is associated with chronic postoperative pain after TKA, but these findings need to be investigated in future studies.

### 4.3 Pain sensitivity in osteoarthritis

Accumulating evidence suggests that severe knee OA pain is associated with widespread pressure hyperalgesia, facilitated TSP and impaired CPM compared with pain‐free individuals (Arendt‐Nielsen, Skou, et al., 2015; Suokas et al., 2012). Furthermore, some studies suggest that specific subgroups of patients are more pain sensitive than others (Arendt‐Nielsen, Egsgaard, et al., 2015; Finan et al., 2013). The assessment of pain sensitivity seems to be important in OA since accumulating evidence links higher levels of pain sensitivity to worse outcome of standard pain treatment such as total joint arthroplasty (Izumi et al., 2017; Kurien et al., 2018; Larsen, Laursen, Edwards, et al., 2021), treatment with NSAIDs (Arendt‐Nielsen et al., 2016; Edwards et al., 2016; Petersen, Olesen, et al., 2019; Petersen, Simonsen, et al., 2019) and exercise therapy (Hansen et al., 2020; O'Leary et al., 2018). It has been argued that maintaining widespread hyperalgesia, facilitated TSP and impaired CPM rely on a peripheral driver (i.e., a prolonged painful input such as an OA affected knee) (Arendt‐Nielsen & Graven‐Nielsen, 2011; Graven‐Nielsen & Arendt‐Nielsen, 2010). Furthermore, studies have reported normalization in PPTs, TSP and CPM in patients who were pain‐free after TKA (Graven‐Nielsen et al., 2012; Kosek & Ordeberg, 2000). More recent studies suggest that not all patients experience normalization in sensitization parameters following total joint arthroplasty (Izumi et al., 2017; Petersen, Arendt‐Nielsen, et al., 2015), and indeed the current study suggests differences in QST profiles in patients with or without chronic postoperative pain after TKA.

Similarly, a subset of patients with OA display low‐grade systemic inflammation (Siebuhr et al., 2014), which is associated with pain sensitization (Schaible, 2014). Thus, it would be logical that the use of NSAIDs should normalize the QST parameters. Arendt‐Nielsen et al. (2016) demonstrated that 4 weeks of 60 mg/day etoricoxib (a COX‐2 inhibitor) might normalize PPTs and TSP, whereas Petersen et al. (Petersen, Simonsen, et al., 2019) demonstrated no modulatory effect of 3 weeks of ibuprofen 400 mg (three times per day) and acetaminophen 1 g (three times per day) on PPTs and CPM. These conflicting results demonstrate a need for more research into understanding the value of QST in OA and how central pain mechanisms can potentially be modulated.

The current study may indicate that postoperative synovitis could be the pain generator which acts as the peripheral driver for postoperatively facilitated TSP. However, future studies are needed to confirm these preliminary results.

### 4.4 Limitations

This exploratory study is limited by the lack of an a priori defined sample size equation and is based on a relatively small cohort of patients. A post hoc sample size estimation revealed that the current study is powered to detect an effect size of approximately 0.8 with a power of 80% and a significant level of 0.05 indicating that only very large differences would appear as findings in the current work. This could indicate that the results for PPT and CPM are under powered in the current analysis, since the effect sizes were below 0.8. Additionally, at least 65 patients are needed for a linear regression model with seven independent parameters as illustrated in this work. Hence, the findings of this exploratory study should be interpreted with care.

Chronic postoperative pain was assessed as the worst pain within the last 24 h, which could be considered a limitation. Future studies should include a broader range of pain assessments such as the Brief Pain Inventory, the Western Ontario and McMaster Universities Osteoarthritis Index or the Knee injury and Osteoarthritis Outcome Score.

The group‐wise comparisons and the correlation analysis reported in the current study are unadjusted for multiple comparison and therefore the results of this exploratory study should be interpreted with care.

The current study only assessed pain catastrophizing as a cognitive factor, but recent studies have demonstrated that assessing symptoms of anxiety and depression might also be important for pain in OA (Larsen, Laursen, Simonsen, et al., 2021; Petersen et al., 2022), and therefore, assessments for anxiety and depression could be implemented in future studies.

## 5 CONCLUSION

This explorative study is the first to report that patients with moderate‐to‐severe chronic postoperative pain show higher scores of synovitis and increased pain sensitivity compared with patients with none‐to‐mild postoperative pain 6 months after total knee arthroplasty. A linear regression model indicates that the degree of synovitis, effusion grade, temporal summation of pain and pain catastrophizing are independent factors associated with the intensity of chronic postoperative pain. These findings indicate that chronic postoperative pain after total knee arthroplasty is multifactorial and that the treatment of chronic postoperative pain should potentially target more than joint‐related pain factors, but larger studies are needed to investigate this.

### AUTHOR CONTRIBUTION

TK, RWK, TG‐N, LA‐N, DPA, KE, BES and KKP conceptualized the design of the study, wrote the protocol and initiated the study. TK, DPA and BES implemented the protocol, communicated with the Regulatory Authorities and conducted the study. TK and KKP analysed the data and wrote the first draft of the manuscript. All authors critically revised the manuscript and approved the final version.

### FUNDING INFORMATION

Center for Neuroplasticity and Pain (CNAP) is supported by the Danish National Research Foundation (DNRF121). The Center for Mathematical Modelling of Knee Osteoarthritis (MathKOA) is funded by the Novo Nordisk Foundation (NNF21OC0065373).

### CONFLICT OF INTEREST

The authors declare no conflict of interest.
